# Conducting epigenetics research with refugees and asylum seekers: attending to the ethical challenges

**DOI:** 10.1186/s13148-021-01092-8

**Published:** 2021-05-08

**Authors:** Faten Taki, Inmaculada de Melo-Martin

**Affiliations:** 1 Department of Anesthesiology, Weill Cornell Medicine, New York, NY USA; 2 Department of Medicine, Division of Medical Ethics, Weill Cornell Medicine, New York, NY USA

**Keywords:** Refugees, Asylum seekers, Epigenetics, Ethical challenges

## Abstract

An increase in global violence has forced the displacement of more than 70 million people, including 26 million refugees and 3.5 asylum seekers. Refugees and asylum seekers face serious socioeconomic and healthcare barriers and are therefore particularly vulnerable to physical and mental health risks, which are sometimes exacerbated by immigration policies and local social discriminations. Calls for a strong evidence base for humanitarian action have encouraged conducting research to address the barriers and needs of refugees and asylum seekers. Given the role of epigenetics factors to mediate the effect of psychological and environmental exposures, epigenetic modifications have been used as biomarkers for life adversity and disease states. Therefore, epigenetic research can be potentially beneficial to address some of the issues associated with refugees and asylum seekers. Here, we review the value of previous and ongoing epigenetic studies with traumatized populations, explore some of the ethical challenges associated with epigenetic research with refugees and asylees and offer suggestions to address or mitigate some of these challenges. Researchers have an ethical responsibility to implement strategies to minimize the harms and maximize the short and long-term benefits to refugee and asylee participants.

## Introduction

More than 70 million people have been forcibly displaced due to the increase in global violence worldwide [1]. Approximately, 26 million of these migrants are refugees. They have crossed international borders and are unable or unwilling to return due to a well-founded fear of persecution based on their race, religion, nationality, political opinion, or membership in a particular social group. Approximately, 3.5 million people are asylum seekers who are seeking sanctuary in another country and have applied for the right to be recognized as a refugee and receive legal protection and material assistance [2].

Refugees and asylum seekers confront difficult social challenges related to finding employment and affordable housing [3], language and communication barriers [4, 5], racism and discrimination [6], and complications dealing with immigration and refugee policies [7]. They also face complex health needs. They are at an increased risk of infectious diseases, experience significant sexual and reproductive health issues, and suffer non-communicable diseases such as hypertension and diabetes that are often poorly managed [8]. Moreover, compared to the broader community in host countries, refugees and asylum-seekers experience higher rates of psychological disorders because of trauma caused by war, persecution, sexual abuse, and torture. Such traumas are compounded with the loss of social networks, the stress of displacement, and the anxieties surrounding integration into countries of settlement.

Governments and humanitarian organizations are calling for more rigorous evidence to develop and implement social and health initiatives for the refugee and asylum-seeking populations and to improve the delivery of emergency services [9, 10]. Thus, there has been an increase in research efforts with the goal of understanding and addressing the unique social and healthcare issues and needs of, and determining the structural barriers encountered by, refugees and asylum seekers. There are legitimate questions about whether epigenetic research should be conducted with vulnerable populations, given the challenges of conducting this research in ethically sound ways [11, 12]. Nonetheless, epigenetic research is being done with these populations and some believe that it can be a valuable research endeavor, particularly in trying to determine the effects of acute war trauma on the physical and mental health of refugee children [13, 14]. Still, conducting research with these populations presents significant ethical challenges. The purpose of this paper is to explore some of the ethical challenges involved in conducting epigenetic research with refugees and asylum seekers. Familiarity with concerns likely to arise when conducting this research is the first step to ensure that studies are performed in ethically sound ways. We also offer some suggestions aimed at addressing some of these ethical challenges.

## Using epigenetics research to advance the needs of refugees and asylum seekers

Genetic, epigenetic, and environmental factors contribute to people’s health status. Epigenetic factors, which act as mediators between genes and the environment, include any process that alters gene activity without changing the DNA sequence. Epigenetic factors such as histone modifications, DNA methylation, and RNA-mediated regulation, allow or inhibit various regulatory molecules to control the expression of genes, which eventually translates to normal or abnormal traits and phenotypes (i.e., healthy or diseased states) [15]. Epigenetic research has attracted a lot of attention in the biomedical and the social sciences. Epigenetic studies are engaging with important epidemiological questions regarding the relationship between health and social status, the role of early developmental events on the health of adults, the influence of lifestyles on people’s phenotype later in life, the health effects of exposures to various environmental chemicals, and the impact of these various factors across generations. Epigenetic modifications have been used as biomarkers for life adversity [16], pregnancy outcomes [17] and reproductive disease [18]. Recent epigenetic research has called attention to the ways in which violence can produce epigenetic changes that can affect physical and mental health status and can transcend generations [14].

Given what is known about the role of epigenetics factors to mediate the effects of different social and environmental contexts across generations [19], many investigators report that epigenetic research can be of great value to refugees and asylum seekers. For instance, ongoing research with these populations attempts to improve our understanding of the contribution of multi-level mechanisms on the mental health of refugee children. Such knowledge could pave the way for the development of protective measures aimed at promoting children’s psychological resilience and reduce the health risks of children impacted by war and forced migration [20, 21]. Epigenetic factors have been shown to act as mediators of resilience and can serve as biomarkers for mental burden [22] and physical health (i.e., birthweight) in populations suffering from war and violence [13, 14, 23]. Moreover, since the biological pathways for childhood vulnerability remain unclear, a better understanding of the contribution of epigenetic factors to stress responses could allow for targeted interventions to prevent the progression of psychiatric diseases [24]. Other studies have identified associations between epigenetic changes in some genes and PTSD severity in populations exposed to genocide violence as well as in their children [25]. Applying epigenetic approaches in refugee settings could provide knowledge regarding biological mechanisms and biomarkers for a potential intergenerational transmission of trauma. Identifying such biomarkers could improve risk stratification, facilitate rapid screening, and promote early intervention to prevent PTSD in children [26]. Linking psycho-behavioral outcomes with epigenetics could help in the development of tailored preventative measures for youth [27] or uncover health disparities long before they emerge [28].

Additionally, the inclusion of epigenetic endpoints in studying the effects of environmental and life conditions on the health of refugees and asylum seekers can help provide quantitative evidence for exposure to adversity and can promote environmental and social justice initiatives [29, 30], improved healthcare services, and transgenerational equity [30]. Epigenetic evidence could thus support calls for action that necessitate protective measures for refugee and asylum seeking women during the antenatal period such as free access to healthcare regardless of the local host policy and special lodging accommodations with access to secure bathrooms, good nutrition, and sanitation. These studies might also be useful to improve refugee women’s reproductive health such as predicting pregnancy outcomes [31, 32], post-partum depression or antenatal mood changes [33, 34], infant’s predisposition to disease [35, 36], inferring exposure to gender-based violence or domestic abuse [37] and supporting asylum applications by providing molecular and quantifiable evidence related to their experiences.

## Conducting epigenetic research with refugees and asylum seekers: ethical issues

Although epigenetic research could benefit refugees and asylum seekers, conducting this research presents significant ethical challenges with which researchers must be unfamiliar. Some of these challenges are not unique to epigenetic investigations and are discussed in widely accepted ethical guidelines such as the Belmont Report [38], and the Declaration of Helsinki [39], which provide ethical principles for clinical research involving human subjects. Nonetheless some research can make some ethical challenges more salient and concerning. This can be the case for epigenetic studies. Take, for instance, the requirement of obtaining informed consent, a requirement grounded on the principle of respect for autonomy [38, 39]. Evidence shows that the quality of the informed consent process in all types of research is often inadequate [40]. Obtaining a free and informed consent from refugees and asylum seekers can be even more difficult because of communication barriers, language proficiency, cultural differences, low health literacy, and insufficient knowledge of research methods and practices [41]. Refugees and asylum seekers’ capacities for autonomy and their ability to provide a free and informed consent can be negatively affected by trauma, displacement, oppression, and the constraining social and political circumstances in which they find themselves. Epigenetic research can compound these problems. Epigenetic results are complex and difficult to interpret. The vast amount of information generated and the difficulty of understanding the relevance and health implications of some of this information can undermine research participants’ ability to provide a truly informed consent in the best of situations. Moreover, epigenetic results often involve risk predictions and this type of information poses further challenges to participants’ ability for autonomous decision-making. Studies show that people incorporate emotions into their decision-making process and regard certain numbers as having affective meaning, particularly when decisions involve risk predictions [42]. Communicating the complexity, risks, potential benefits, and uncertainty of epigenetic information and delivering this information to individuals from different cultural or language backgrounds and who often have varying levels of health literacy [43] presents additional worries regarding whether refugees and asylum seekers can provide an autonomous authorization. Similarly, biological samples need to be collected to conduct epigenetic research, which can clash with various cultural and religious beliefs. Differences in regulatory frameworks regarding ownership of bodily specimens pose additional comprehension barriers by refugees and asylum seekers.

Autonomous authorization can be threatened not only by inadequate disclosure of information or insufficient comprehension, but also by various pressures, manipulation, or undue inducements. A valid consent to research must not only be informed, but also free [38, 39, 44]. Poverty, psychological and physical trauma, legal-status uncertainty, and pressing medical needs make refugees and asylum seekers particularly vulnerable to voluntariness threats. Although compensation incentives might be appropriate given the social and financial conditions that these populations face, these incentives can also work as undue inducements precisely because of those conditions. Thus, although concerns about exploitation and undue inducement are present in other types of research contexts, the circumstances under which research with refugees usually happen, necessitate even a more careful attention to what incentives to provide.

Similarly, maximizing possible benefits of participation is an important ethical goal [38, 39]. However, researchers’ desires to ensure that the results of their investigations can lead to development of policies likely to enhance the plight of refugees can also threaten the voluntariness of potential participants [45]. For instance, researchers might present such potential benefits with more enthusiasm than might be warranted. This concern is particularly salient when conducting epigenetic research, given the novel nature of much of the technology. Indeed, in some instances, epigenetic research results or their policy implications have been overstated [46]. Translating multi-faceted societal and environmental adversities to molecular epigenetic codes can be more difficult than sometimes researchers acknowledge [43].

When conducting research, investigators are also required to minimize risks of harm to participants and to balance such risks in relation to potential benefits to participants or to society [38, 39]. Any type of research conducted with refugees and asylum seekers needs to be attentive to conditions that can increase possible harms to participants [47]. But certain research topics and methodologies can place members of these populations at increased risk of harm because of social stigma or discrimination. This could be the case for epigenetic research. As indicated earlier, epigenetic markers provide information about exposure to environmental factors, lifestyle decisions, or adversity. This information can potentially harm refugees and asylum seekers in two different ways. First, participants can be held responsible for at least some aspects of their health risks and accused of burdening the economy of recipient countries, thus further stigmatizing these groups [48]. Second, the information gathered could be used to discriminate against refugees and asylum seekers in various ways, including during legal proceedings [49]. Recent controversies over the application of epigenetic age estimation in forensic context to assess young migrant refugee’s age [50] calls attention to the detrimental effects that some epigenetic information can produce. Such research carries the risk of inaccurately estimating the biological age of youth due to the inherent technical aspects associated with epigenetic technology and the lack of reference groups. Moreover, trauma, stress, and psychiatric conditions can accelerate epigenetic ageing [51], which can prevent refugee youth of unknown age from accessing certain benefits in the host countries.

Despite the potential for the development of therapeutics from epigenetics studies, refugees and asylum seekers might be unable to afford treatments that can reverse the negative health effects [30]. When research fails to result in improvements for the lives of research subjects and their communities, it can lead participants to feel that researchers are taking advantage of them, that their participation is useless, and that their voices are not being heard. This situation can also harm participants’ sense of self, contribute to their traumas, and can undermine trust in the research community [52].

Confidentiality protections, another basic ethical requirement [38, 39], can likewise be threatened in contexts with insufficient security safeguards and lack of private spaces such as refugee camps. Epigenetic research with refugees and asylum seekers may heighten concerns about privacy and confidentiality. First, profiling the epigenome can involve an unprecedented level of exposure of personal information as it can inform not only about physical and mental health risks, but also about a person’s travel history [53], sexual behavior [54], and drug use [55]. Second, at least some epigenetic techniques could expose genetic information and permit the reidentification of sample donors [56]. Inadequate protection of this data is a concern for any research subject, but it is even more problematic when refugees and asylum seekers are involved, given that such information can put their lives and wellbeing at risk and threaten legal proceedings. Doubts about whether existing legal frameworks and encryption mechanisms, designed to protect the privacy of individual genetic information, can adequately protect individual epigenetic data further increases concerns about ensuring the security of biological information for these vulnerable populations [11]. Third, low- or middle-income host countries often lack the technology to process the samples for epigenetic analysis, which require the samples to be shipped to other countries. This can increase confidentiality risks.

## Addressing challenges of conducting epigenetic research with refugees and asylum seekers

Addressing the challenges of conducting epigenetic research with refugees and asylum seekers is not straightforward due to the inherent research conditions and the vulnerabilities of refugees and asylum seekers. Still making this task more difficult is the fact that although existing ethical codes such as the Belmont Report or the Declaration of Helsinki provide ethical principles to guide research with human subjects, these codes often require interpretation, are sometimes vague about recommendations, and do not explicitly address research with refugees or asylum seekers. Indeed, even when significant agreement exists that some research participants are vulnerable and in need of special safeguards [38, 39], there are disagreements about who exactly is vulnerable, what grounds vulnerability, and how to best protect vulnerable groups [57–59]. Nonetheless, researchers have a responsibility to try to minimize possible harms to research participants by developing strategies to tackle the ethical challenges they are likely to face.

To create conditions that are more likely to allow refugees and asylum seekers to provide autonomous authorization, researchers can develop strategies to minimize communication barriers. Engaging community members in the design of the research and the goals of the investigation can give researchers clues about possible communication barriers, and about important versus unacceptable issues to potential participants.

Ensuring appropriate time with participants to understand necessary information and follow up with questions is obviously important; however, researchers need to ensure that the materials used to recruit and inform potential participants are also adequate. To achieve this goal, researchers can incorporate science communication training in the research design [60]. Collaborations with educators and local groups that are familiar with relevant cultural backgrounds can help in delivering engaging and culturally sensitive information about the risks and potential benefits of the research conducted. Such collaborations could aid researchers in providing appropriate information regarding controversial topics such as ownership of specimens and treatment of bodily samples. Also, the use of decision-aids can facilitate understanding and enhance the quality of the informed consent process. Appropriately developed decision-aids can improve knowledge of key aspects of a decision, clarify perceptions of the probabilities of various outcomes, and help determine whether there is a match between preferred outcomes and the choice participants make [61]. Such aids can be particularly useful in the context of complex, predictive results such as that involved in epigenetic research [62].

Addressing concerns about the need to maximize potential benefits of epigenetic research requires researchers to accurately describe such benefits and ensure the participants’ voluntariness by avoiding undue inducements. It also requires that researchers balance the risks and potential benefits of participation. At this point, epigenetics research is unlikely to offer a direct benefit to participants. Potential benefits of epigenetic research would accrue to future refugees and asylum seekers and to society in general. Thus, researchers should find other ways to benefit those who consent to participate in epigenetic research. For instance, investigators could collaborate with local partners and community groups to gather information about some of the needs of refugees and asylum seekers being recruited. Local partners and community members are likely to have nuanced and culturally sensitive knowledge of what types of benefits could be appropriate to offer and researchers could then budget for some of them. Establishing collaborations with local groups, which can include cooperatives within the refugee camps, can encourage knowledge sharing and training for specific skillsets and tool handling [63]. Educational awareness programs, skill training, health education classes could all enhance participants’ experiences, promote their wellbeing, and enhance sustainability [9]. These types of measures are also unlikely to threaten refugees’ and asylum seekers’ ability to provide voluntary decisions.

One of the main potential harms of conducting epigenetic research with refugees and asylum seekers arises from privacy and confidentiality risks. To protect research participants against unauthorized disclosure of confidential information, researchers must ensure the strictest measures regarding handling, storage, and access to epigenetic specimens and data. As indicated, epigenetic information can be used to discriminate against refugees and asylum seekers, negatively affect legal proceedings cases, and prevent them from access to social services [11]. De-identifying and unlinking personal information from epigenetic samples, especially given that much of it does not constitute actionable data, should be done in order to protect participants’ privacy rights. In contexts where government agencies have extensive powers to access people’s information, researchers should consider not conducting epigenetic research, if the confidentiality of epigenetic data cannot be reasonably ensured as the use of such data can produce great harms to refugees and asylum seekers [12] that are unlikely to be balanced by potential benefits.

## Conclusion

Although there are still important disagreements about whether it is ethically appropriate to conduct epigenetic research with refugees and asylum seekers at this time [11, 12], epigenetic research could become valuable in trying to address the social and health needs of an increasing numbers of refugees and asylum seekers. Nonetheless, conducting epigenetic studies involves various ethical challenges that are made more difficult because of the nature of such research and because of the vulnerabilities affecting these populations. Researchers thus have an ethical responsibility to ensure that they are familiar with the problems they are likely to confront and that they have implemented strategies to minimize potential harms and maximize possible benefits to the participants and to society. But concerns about the vulnerabilities of these populations should not deter investigators from pursuing important research questions with refugees and asylum seekers. Disproportionate attention to the difficulties endured by these populations can lead to a lack of appreciation for their resilience. It can thus harm these populations by excluding them from the potential benefits that research can bring. Collaborations between researchers and migration advocacy groups are needed to develop refugee-specific ethical guidelines to conduct epigenetic research with this population.
